# A Simple yet Accurate Method for the Estimation of the Biovolume of Planktonic Microorganisms

**DOI:** 10.1371/journal.pone.0151955

**Published:** 2016-05-19

**Authors:** Alessandro Saccà

**Affiliations:** Department of Biological and Environmental Sciences, University of Messina, Messina, Italy; University of Lincoln, UNITED KINGDOM

## Abstract

Determining the biomass of microbial plankton is central to the study of fluxes of energy and materials in aquatic ecosystems. This is typically accomplished by applying proper volume-to-carbon conversion factors to group-specific abundances and biovolumes. A critical step in this approach is the accurate estimation of biovolume from two-dimensional (2D) data such as those available through conventional microscopy techniques or flow-through imaging systems. This paper describes a simple yet accurate method for the assessment of the biovolume of planktonic microorganisms, which works with any image analysis system allowing for the measurement of linear distances and the estimation of the cross sectional area of an object from a 2D digital image. The proposed method is based on Archimedes’ principle about the relationship between the volume of a sphere and that of a cylinder in which the sphere is inscribed, plus a coefficient of ‘unellipticity’ introduced here. Validation and careful evaluation of the method are provided using a variety of approaches. The new method proved to be highly precise with all convex shapes characterised by approximate rotational symmetry, and combining it with an existing method specific for highly concave or branched shapes allows covering the great majority of cases with good reliability. Thanks to its accuracy, consistency, and low resources demand, the new method can conveniently be used in substitution of any extant method designed for convex shapes, and can readily be coupled with automated cell imaging technologies, including state-of-the-art flow-through imaging devices.

## Introduction

The problem of estimating the biomass of microbial plankton is paramount to any ecological study concerned with fluxes of energy and materials in an aquatic ecosystem, and has been addressed many times [1–17]. Cell counts *per se* are inadequate as a measure of microbial biomass [18, 19] since in a microbial community, microorganism size spectrum encompasses several orders of magnitude, and even within lower taxa—such as genera and species—size variability may be significant. Microbial biomass is thus estimated from biovolume, i.e. the extent of space occupied by a cell in its environment. A volume-to-carbon relationship is then applied to determine the amount of organic matter, in carbon units, to be ascribed to organisms of a certain taxon, size class, trophic level, etc., that are present in a given unit space of their environment.

Cell biovolumes are typically estimated from digitised microscope pictures via image analysis, a powerful tool enabling the extraction of relevant information from 2D images in view of a few basic assumptions, which allow for the handling of the cells as if they were simple solids of revolution, obtained by rotating a plain, convex surface around a straight axis. The simplest approach consists of measuring a few basic parameters such as the putative symmetry axis of the cell and its widest perpendicular distance—or, alternatively, the cross-sectional area and perimeter of the cell—and in calculating the volume of either a prolate spheroid or a cylinder with hemispherical ends (the latter is preferable for prokaryotes) through a single equation or a small set of Eqs 1–6.

To overcome the limitation of assuming approximate rotational symmetry, other methods have been introduced that are based on standardised sets of geometric shapes [7–14]. A simple or composite geometric shape is attributed to any consistently-shaped microbial taxon, and the biovolume of each cell is calculated from a small number of linear dimensions. The outline of a microorganism, however, often departs from that of any conceivable combination of a few basic shapes, as it shows bulges, concavities, grooves and other features—especially in eukaryotes—that are overlooked by this approach. This method, in addition to requiring a competent taxonomist who is acquainted with the morphology of a number of microbial taxa, is time-consuming and cannot be readily automated.

Again, to circumvent these issues, a few computational methods have been proposed for the estimation of the biovolume of planktonic microorganisms [15–17]. Their common rationale consists in automatically producing a precise 3D reconstruction of the cell shape from its 2D outline through iterative algorithms. These methods, however, must be carefully designed to avoid any incoherent results due to unexpected cell shapes, and should not be intensive in terms of computational resources. In fact, all of these methods require relatively complex pre-processing for proper edge detection and identification of closed boundaries. Among these methods, only the ‘distance map’ algorithm [17] is specifically intended for eukaryotes (in the size range of microplankton), but the developers of this method advise using it in combination with an ‘integration’ algorithm [15], given the superior performance of the latter with plain, convex shapes and its higher computational efficiency [17].

The cutting-edge in plankton ecology studies is represented by combinations of automated sampling devices, image analysis technologies and machine learning algorithms that allow for the counting and sizing of plankton organisms quite rapidly and effortlessly (see [20] for a review). The strength of these automated methods lies in their speed, allowing for larger scale and/or finer structure analyses than is practical manually. Although the reliability of the data provided by these automated methods is still under assessment, they are bound to replace manual or semi-automated ones in many ecological studies, provided that they can rely on a viable method for biovolume estimation.

Bearing in mind that it is unrealistic to determine the exact biovolume of a microorganism from a single 2D picture, a new method has been devised that is both accurate and versatile. What’s more, this method neither impacts human labour, nor computational resources, and can also be readily automated.

## Materials and Methods

### Method rationale

A well-known Archimedes’ principle states that the volume of a sphere is equal to two-thirds the volume of the cylinder in which the sphere is inscribed. In truth, this is valid not only for a sphere, but also for any regular ellipsoid, the equation for which (with standard axis-alignment) in a *xyz*-Cartesian coordinate system is:

$$\frac{x^{2}}{a^{2}}+\frac{y^{2}}{b^{2}}+\frac{z^{2}}{c^{2}}\leq 1$$ (1)

where *a*, *b* and *c* are fixed positive real numbers determining the aspect ratios of the ellipsoid.

The volume of the cylinder in which the ellipsoid is inscribed is:

V=πab*2c (2)

where (π *a b*) is the surface area of the elliptic cylinder base and (2 *c*) is the height of the cylinder (S1 Fig). If we multiply this expression by 2/3, the outcome, i.e., the volume of the ellipsoid, is:

$$V=\frac{4}{3}\;\pi \;a\;b\;c$$ (3)

This means that it is possible to calculate the volume of any regular ellipsoid if the basic figures of the cylinder in which it is inscribed are known. Instances of ellipsoids include, as particular cases, those of prolate and oblate spheroids—obtained by rotating a half-ellipse around one of its principal axes—and, by extension, that of a sphere. In other words, the equation for calculating the volume of any regular ellipsoid is:

$$V=\frac{2}{3}A\;d$$ (4)

where *V* is the volume of the ellipsoid, *A* is the surface area of the elliptic cross-section containing two out of the three principal axes, and *d* is the axis of the ellipsoid perpendicular to this elliptic cross-section. The above formulation is of little advantage in calculating the volume of regular ellipsoids, but is of great value for volume estimation of irregular, though roughly ellipsoidal, objects.

**Fig 1:**
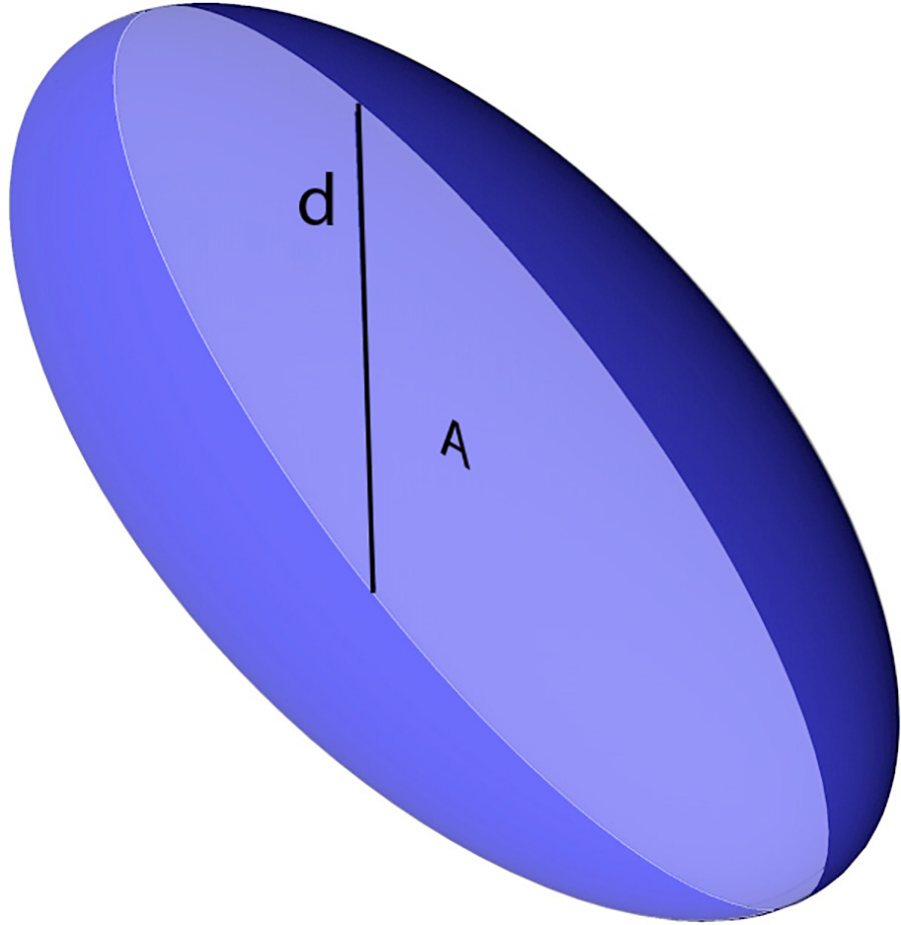
Half-transparent 3D model of a cell featuring the shape of a prolate spheroid. *A* is the cross-sectional surface area and *d* is the cell width, which is equal to the obscured third dimension due to the assumed rotational symmetry.

Given the assumed rotational symmetry of the cell, its concealed dimension (*d*) can be approximated conveniently by the maximum visible linear dimension perpendicular to the putative symmetry axis (cross section’s width), while *A* corresponds to the surface area of the cell cross section as captured by an image acquisition system (Fig 1). Both parameters can readily be measured through image analysis of digitised micrographs.

This is a significant improvement *per se*, but a further step forward can be made by multiplying the outcome by a *coefficient of unellipticity* (*U*), introduced here for the first time, which accounts for the impact on the whole 3D shape of the divergence of the cell cross-section from a regular ellipse. The coefficient *U* is defined as the square root of the proportion between the surface area of the cell cross-section (*A*) and that of an ellipse (*E*), the principal axes of which being the putative symmetry axis of the cell cross section and its maximum perpendicular distance (proxies for cell length and width, respectively, in elongated cells):

$$U=\sqrt{\frac{A}{E}}$$ (5)

**Fig 2:**
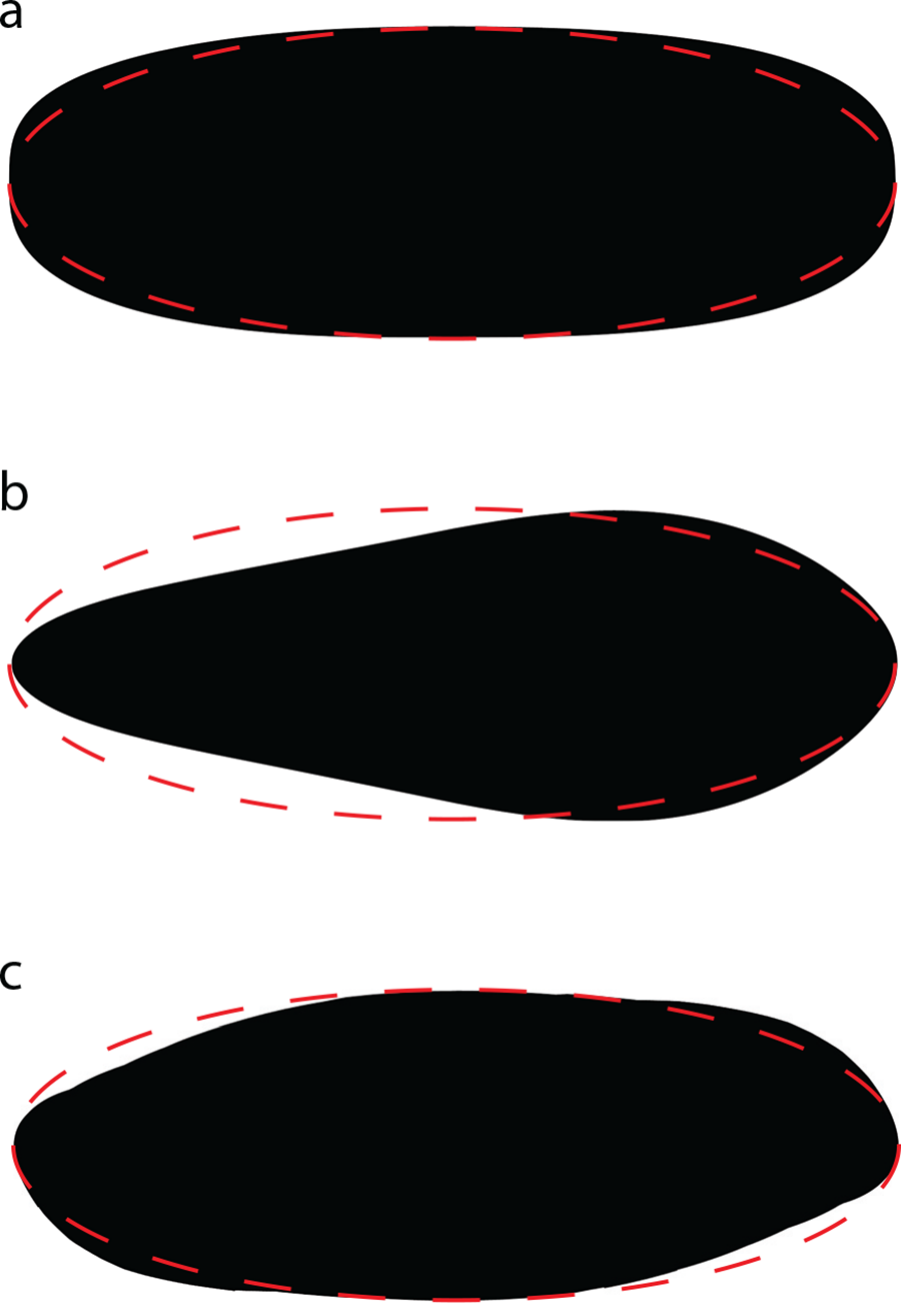
Examples of cell cross sections with different *coefficient of unellipticity (U)*. (**a**) *U* > 1, (**b**) *U* < 1, (**c**) *U* = 1. Solid black silhouettes: cell cross sections; red dashed lines: regular ellipses having the same length and width as the cell cross section.

Of course, the closer the two surface areas, the more the value of *U* is close to 1. For example, if the cross-section is more convex than the ellipse having the same dimensions, then *U* is higher than one (Fig 2A), if it is less convex, then *U* is lower than 1 (Fig 2B). As particular cases, if the cell cross section is a regular ellipse or a sphere *U* is equal to 1, but there can also be irregular shapes for which *U* = 1 (Fig 2C). The estimated volume of a roughly ellipsoidal object is thus:

$$V=\frac{2}{3}A\;d\sqrt{\frac{A}{\frac{\pi \;d\;l}{4}}}$$ (6)

$$V=\frac{4}{3}\;A\;d\sqrt{\frac{A}{\pi \;d\;l}}$$ (7)

and the final equation is:

$$V=\frac{4}{3}\;A\sqrt{\frac{d\;A}{\pi \;l}}$$ (8)

where *l* is the measured cell length (the putative symmetry axis). Clearly, the volume increases significantly with an increase in the surface area of the cell cross-section and, to a lesser extent, with a decrease in the cell aspect ratio (*l*/*d*). When *A* is a circle with radius equal to *d*, and hence *d* is equal to *l*, the volume is that of a sphere.

### Method evaluation

**Fig 3:**
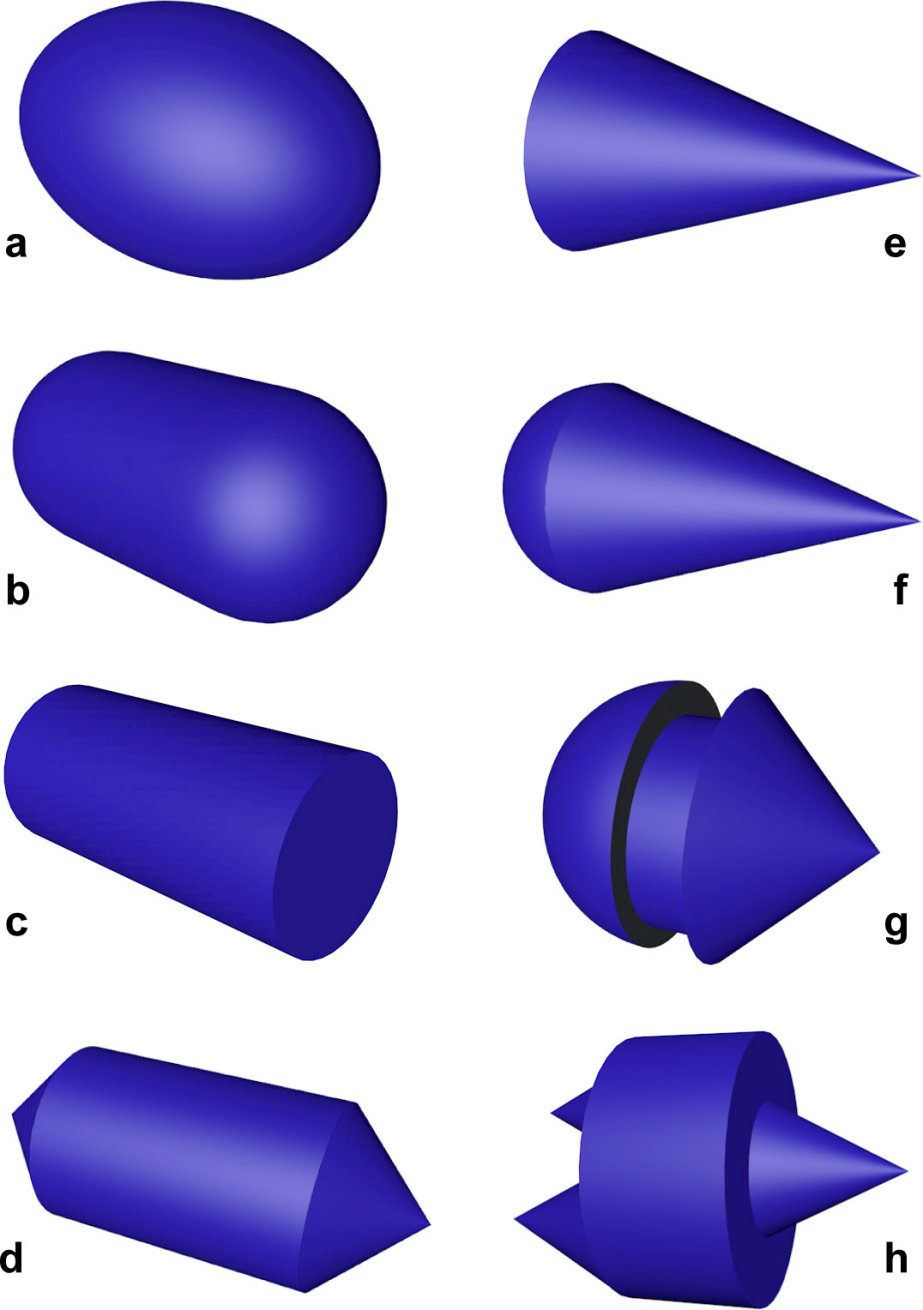
Simple or composite geometric shapes used as models for testing the accuracy of the proposed method. (**a**) prolate spheroid, (**b**) cylinder with hemispherical ends, (**c**) cylinder, (**d**) cylinder with conical ends, (**e**) cone, (**f**) cone with a hemispherical end, (**g**) *Peridinium*-like, (**h**) *Ceratium*-like. The latter two shapes are named after two common microalgae genera.

As both the volume and the cross-sectional area of any regular geometric shape can be calculated exactly, the accuracy and reliability of the proposed equation was tested on several simple or composite geometric shapes reminiscent of common plankton microorganisms (Fig 3 and S2 Fig): prolate spheroid, cylinder with hemispherical ends, cylinder, cylinder with conical ends, cone, cone with a hemispherical end, *Peridinium*-like and *Ceratium*-like (the latter two shapes named after two well-known planktonic protist genera). The proportions of the test shapes were varied across 1,000 regular discrete intervals encompassing an aspect ratio range consistent with that typical for most planktonic microorganisms (between 1:1 and 1:5), and errors in volume estimation were compared to those obtained with the aforementioned methods proposed for prokaryotes [2–6] (subsequently referred to as Fry, Bjørnsen, Bloem, Massana and Blackburn, respectively), as well as to those obtained with the equation for the volume of a prolate spheroid, which is considered suitable e.g. for simple shaped nano-eukaryotes [21].

The proposed equation was also compared with an ‘integration’ approach, following the same basic principle as an algorithm already described [15]. The volume of a single sphere, two contiguous spheres (possible models respectively for cocci and diplococci) and a cylinder with hemispherical ends (the typical model for bacillus-like bacteria)—the latter having an aspect ratio equal to two—were estimated with both methods. In each case, the cross section of every hemisphere was rendered by a circle with radius equal to 1, which was segmented into 10 parts, each 0.1 units wide. Thanks to trigonometric relationships, the lengths *h*_*1*_*…h*_*10*_ of each segment were computed and the volumes of 10 circular cylinders with height 0.1 and radii respectively equal to *h*_*1*_*…h*_*10*_ were calculated. The length of a segment is equal to tan *α*, while *α* is equal to cos^−1^ *x* (where *x* varies between -0.9 through 1 by 0.1 intervals). Being the width of a segment equal to 0.1, the volume of each cylinder is equal to 0.1 tan cos^−1^ *x* and the total volume, according to the ‘integration’ method, amounts to $0.1\;\sum_{1}^{10}{\text{tan cos}}^{-1}\;x$. The impact of the length of the cylindrical part of the shape was also explored.

Comparisons with the integration approach were also performed employing two solids of revolution representing alternative types of cell extremities, and obtained by rotating respectively the planar curves:

$$a)\;Y=X^{2}\;;\;0\leq y\leq h$$ (9)

and

$$b)\;Y=X^{4}\;;\;0\leq y\leq h$$ (10)

around the *y* axis (S3 Fig).

The number and position of sample points used for the ‘integration’ method was varied so as to obtain seven sets of equidistant points (5, 10, 20, 30, 40, 50, and 100 points, respectively). In order to test the variability of the volume estimation accuracy in response to the different position of points, the points in each set were shifted altogether, yielding 11 different subsets for each set. Actual volumes and cross-sectional surface areas of the solids of revolution were determined by integral calculus. All calculations were made using Microsoft® Excel (Microsoft® Office Professional Plus 2010).

**Fig 4:**
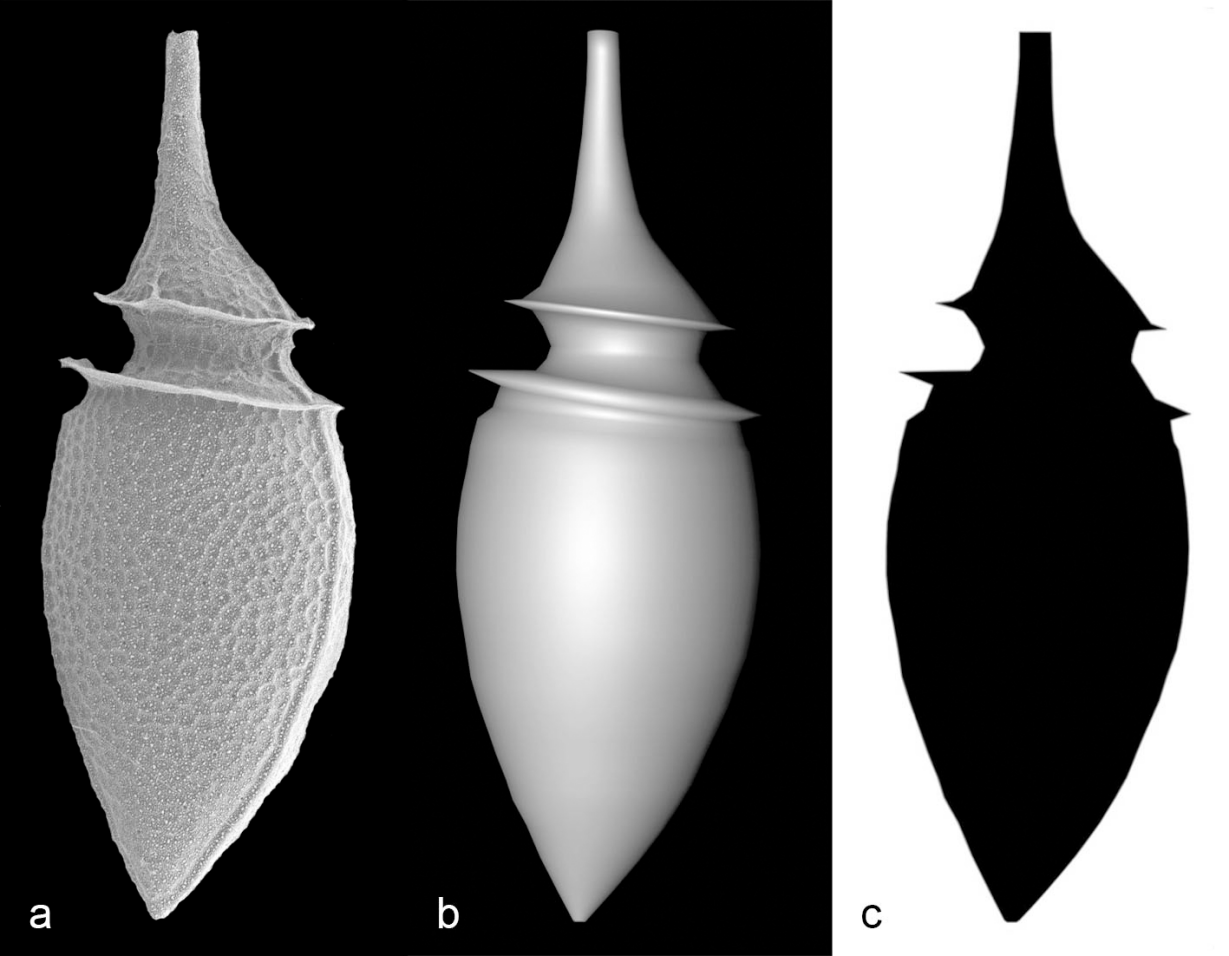
3D model of *Oxyphysis oxytoxoides*, a heterotrophic dinoflagellate species common in temperate to subtropical waters. (**a**) Scanning Electron Microscope picture of an individual of *Oxyphysis oxytoxoides*, (**b**) Binary picture obtained through image segmentation of **a**, and employed for the estimation of the cross-sectional surface area of the cell, (**c**) 3D reconstruction of a cell of *O*. *oxytoxoides* using **a** as a model.

A more realistic test was performed with a 3D model of a relatively complex-shaped phytoplankton cell, such as that of the dinoflagellate species *Oxyphysis oxytoxoides*. Its 3D reconstruction was obtained through the software Cinema4D, version R12 (Maxon Computer GmbH) and was modelled on a 2D picture of the organism (Fig 4) assuming the maximum *z*-dimension of the cell to be equal to the longest cord perpendicular to the major axis on the *xy* plane. Three image resolutions, each with four different *length/width* proportions were considered by changing the length of the model shape (respectively *0*.*5x*, *1x*, *2x*, *3x* the original length). Twelve black and white images were thus created with Photoshop version 12.0 (Adobe Systems Incorporated) starting from orthogonal renderings of the respective 3D models obtained with the built-in function of Cinema4D. If they were pictures of plankton cells captured with Imaging FlowCytobot [22], their measures—21–182 μm equivalent spherical diameter (ESD)—would have encompassed a great part of the microplankton size range and possible aspect ratios.

The volumes of the 3D models were assessed by the newly proposed equation, the prolate spheroid equation, and the methods by Fry, Blackburn, Bjørnsen, Bloem and Massana [2–6] using the maximum and minimum Feret’s diameters, as well as the surface area of the shapes’ cross-sections estimated through the application FIJI (ImageJ 1.49m), a public domain Java-based image processing application developed at the National Institutes of Health (Bethesda, Maryland, USA). Image segmentation, which preceded the measurement stage, was obtained by converting the *xy* view of the 3D object from RGB to greyscale, and then adjusting the ‘input levels’ of the image with Photoshop until the target object was almost entirely black on a white background. The ‘make binary’ function of FIJI was used to convert the few remaining grey pixels into either white or black ones. The actual volume was calculated via the Cinema4D plugin MeshInfo v0.5.0 (Kuroyume's DevelopmentZone). The possible error in volume estimation caused by MeshInfo was evaluated as follows: a set of 30 cylinders with hemispherical ends was constructed with Cinema4D, varying their length from one time (sphere) to 30 times the width. The volume of each cylinder, as estimated through Meshinfo, was compared to the value calculated using Microsoft® Excel.

The cells’ cross sections were also fed to the software YABBA [16], which returned biovolume estimations according to several methods: an original algorithm, henceforth referred to as Zeder [16], as well as Fry, Bloem, Blackburn [2, 4, 6], and an implementation of the ‘integration’ approach [15]. The ‘distance map’ algorithm [17] and an implementation (devised and coded by Heidi Sosik) of the ‘integration’ algorithm were also tested, the latter incorporating special improvements designed to deal with relatively complex shapes, namely those displaying more than one crossing of the target along a slice (Sosik, personal communication). Image processing and all calculations with the latter two approaches were kindly performed by Heidi Sosik with MATLAB (The MathWorks, Inc.).

The same test as that performed on the *O*. *oxytoxoides* model was conducted on a regular prolate spheroid, as well as on a curved cylinder with hemispherical ends, again with three image resolutions and four aspect ratios. Three branched, or otherwise concave, shapes resembling different phytoplankton species were also considered—one of them modelled on the silicoflagellate *Dictyocha fibula*, a species characterised by prominent spiny processes, and the others modelled, respectively, on two colony-forming diatom genera, *Thalassionema* and *Thalassiosira*. None of these models was absolutely realistic, as ‘local’ rotational symmetry was always assumed, even where not present in nature (S4 Fig).

## Results

**Fig 5:**
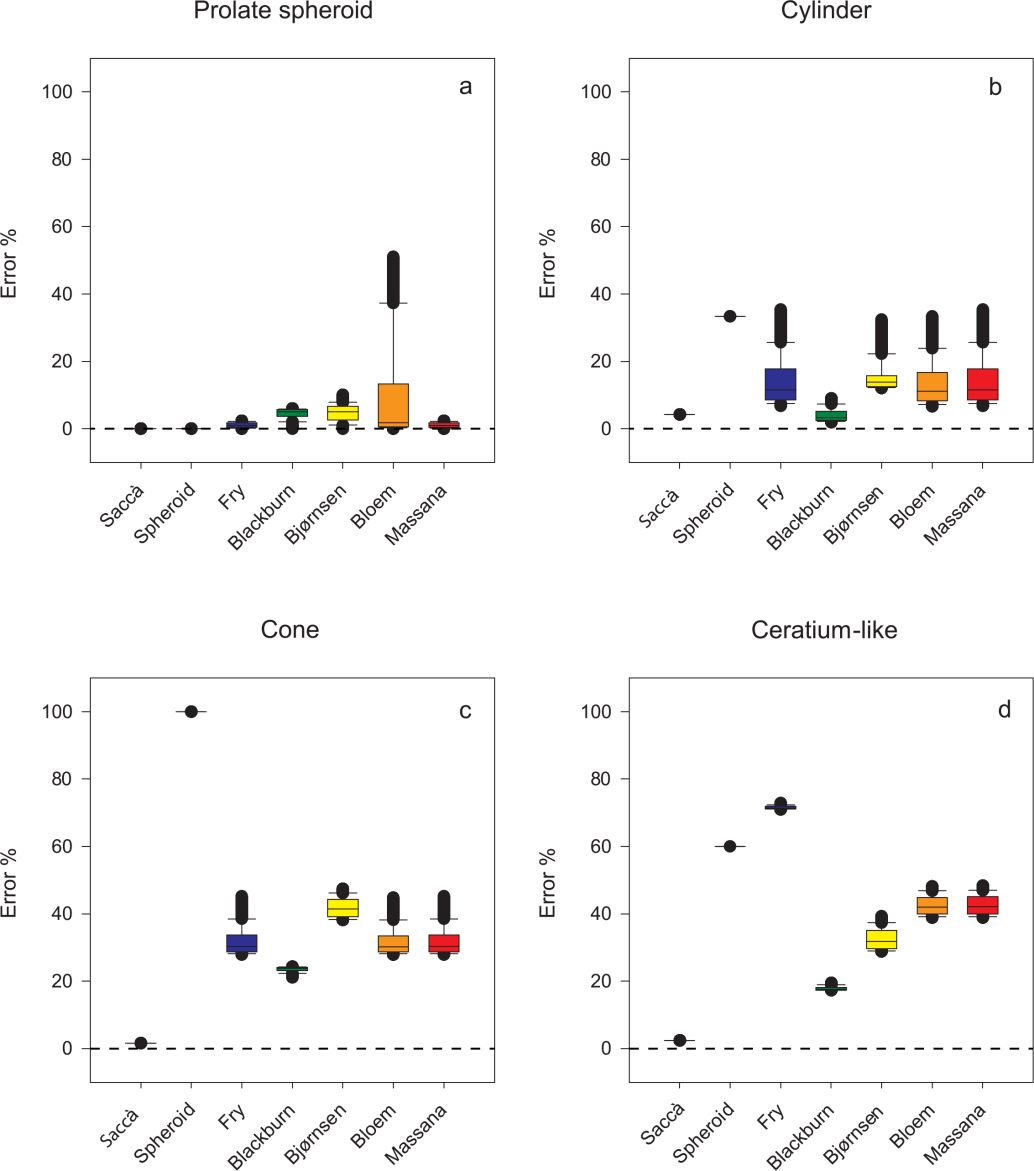
Box plots showing the errors caused by the new equation and the other tested methods in the estimation of the volume of four of the eight simple or composite geometric shapes (those yielding constant errors with the new equation and the prolate spheroid equation). The median, as well as the 10^th^, 25^th^, 75^th^, and 90^th^ percentiles are shown. Whiskers indicate the 10^th^ percentile and 90^th^ percentile, respectively; while the extent of outlying points (solid circles) identifies the data range (standard method is used to calculate percentile values).

**Fig 6:**
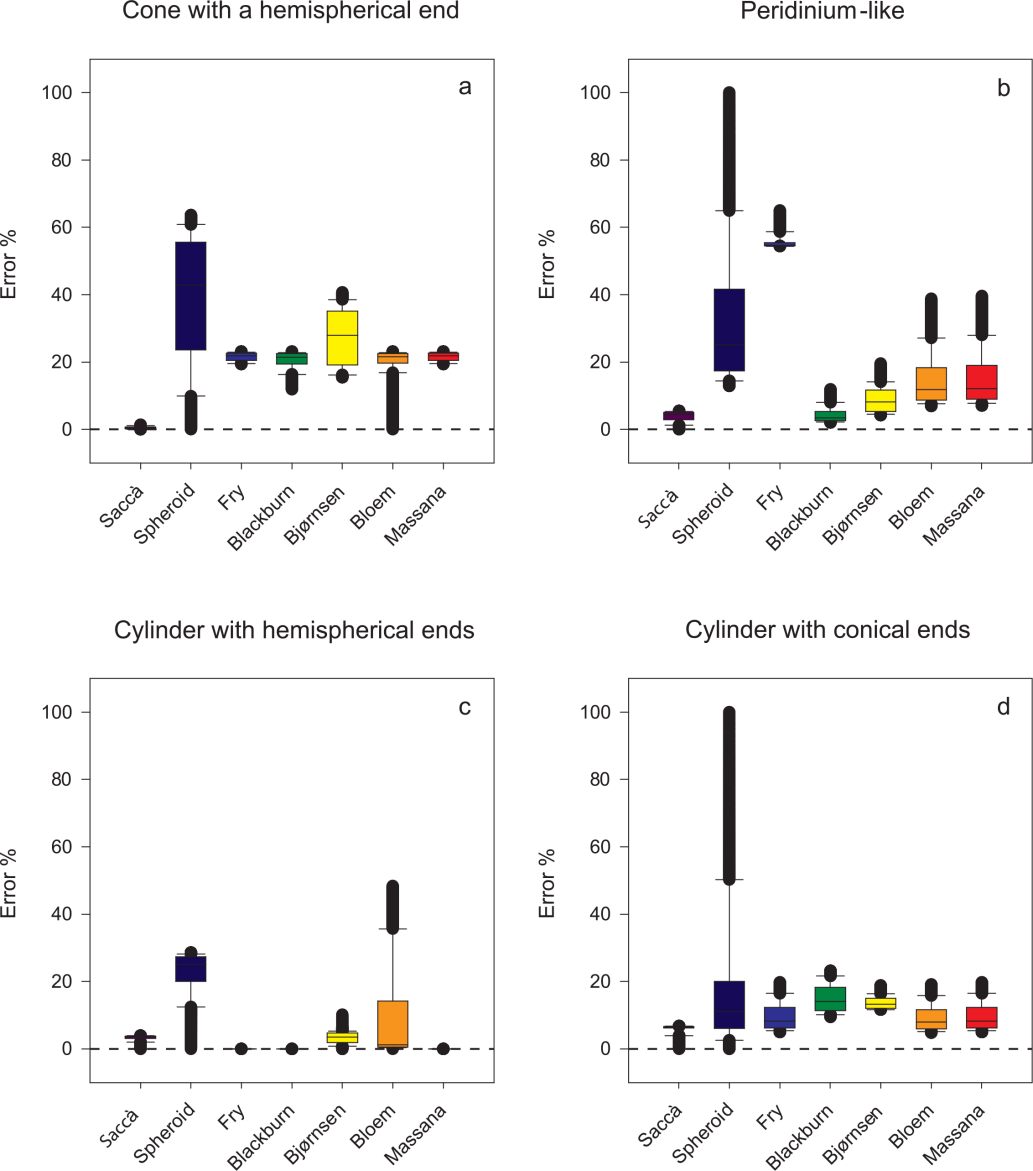
Box plots showing the errors caused by the new equation and the other tested methods in the estimation of the volume of four of the eight simple or composite geometric shapes (those yielding variable errors with the new equation and the prolate spheroid equation). The median, as well as the 10^th^, 25^th^, 75^th^, and 90^th^ percentiles are shown. Whiskers indicate the 10^th^ percentile and 90^th^ percentile, respectively; while the extent of outlying points (solid circles) identifies the data range (standard method is used to calculate percentile values).

In four out of the eight regular geometric shapes, the percentage error was invariable with both the new equation and the prolate spheroid equation, regardless of the aspect ratio; for prolate spheroid, cylinder, cone, and *Ceratium*-like shapes, the errors were, respectively, 0.00%, -4.22%, 1.59%, and 2.41% with the new equation, and 0.00%, 33.3%, 100%, and 60.0% with the prolate spheroid equation (Fig 5). The methods by Fry, Blackburn, and Massana yielded exact results with cylinders with hemispherical ends independent of their aspect ratio, while the new equation returned errors between 0.00% and -3.91% (mean: -3.26%; std. dev.: 0.82%). With all other shapes the proposed equation yielded consistently smaller errors—and narrower variances—than the other six methods, the highest error being 6.68% with cylinder with conical ends (Fig 6).

The comparison between the proposed equation and the ‘integration’ method yielded different results depending on the shape tested (Table 1). In the cases of a single sphere or a double sphere, the error with the ‘integration’ method was equal to -0.25%, while with the proposed method, the biovolume estimation was exact (*U* was equal to 1). The test with a cylinder with hemispherical ends, conversely, returned an error of only -0.10% with the ‘integration’ method and an error of -3.06% with the proposed equation.

**Table 1:** Comparison between the “integration” method by Sieracki et al. (1998) and the newly proposed equation on a single sphere, a double sphere, or on a cylinder with hemispherical ends.

	*Actual Volume*	*Volume Sieracki*	*Volume Saccà*	*Error Sieracki*	*Error Saccà*
Single sphere	4.19	4.18	4.19	-0.25	0.00
Double sphere	8.38	8.36	8.38	-0.25	0.00
Cylinder with hemispherical ends	10.47	10.46	10.15	-0.10	-3.06

Volume is in generic units, while error is in percentage with respect to actual volume.

The proposed equation overestimated the volume of the solid of revolution derived from the curve *Y* = *X*^2^ by 4.27%. A smaller error—an overestimation of 2.80%—was obtained with the proposed equation when tested on the solid of revolution derived from the curve *Y* = *X*^4^. The ‘integration’ method, on the other hand, gave different errors in estimating the volume of each shape, depending on the number and position of sample points (Table 2). With both shapes, a minimum of 20 sample points was needed to obtain, on average, lower errors with the ‘integration’ algorithm than with the newly proposed method, and at least 30 points for certainty (Table 2).

**Table 2:** Percent errors obtained with the ‘integration’ algorithm (Sieracki et al. 1998) in the estimation of the biovolumes respectively of the solids of revolution *a* and *b* (see text).

*Shape*	*Number of points*	*Min*	*Max*	*Mean*	*Std*. *Dev*.
*a*	5	0.00	20.00	*10*.*91*	*6*.*71*
	10	0.00	10.00	*5*.*45*	*3*.*36*
	20	0.00	5.00	*2*.*73*	*1*.*68*
	30	0.00	3.33	*1*.*82*	*1*.*12*
	40	0.00	2.50	*1*.*36*	*0*.*84*
	50	0.00	2.00	*1*.*09*	*0*.*67*
	100	0.00	1.00	*0*.*55*	*0*.*34*
*b*	5	0.00	17.54	*7*.*27*	*5*.*04*
	10	0.00	8.42	*3*.*29*	*2*.*06*
	20	0.00	4.08	*1*.*92*	*1*.*24*
	30	0.00	2.68	*1*.*30*	*0*.*83*
	40	0.00	1.99	*0*.*98*	*0*.*62*
	50	0.00	1.59	*0*.*82*	*0*.*57*
	100	0.00	0.78	*0*.*40*	*0*.*25*

Seven sets of points of different size were tested for each shape, and means and standard deviations were calculated over eleven different point displacements for each set (n = 11): the optimum (error = 0.00%) plus five shifted to one side of the optimum and five to the other side.

Meshinfo always underestimated the volume of cylinders with hemispherical ends. The error caused by Meshinfo decreased progressively with shape length, namely between 1.46% (sphere) to 0.53% (maximum length), with a mean of 0.61% and a standard deviation of 0.18%. These discrepancies being relatively small, and considering the unpredictability of the behaviour of Meshinfo with different shapes, no attempt was made to correct for actual volume misestimation.

**Fig 7:**
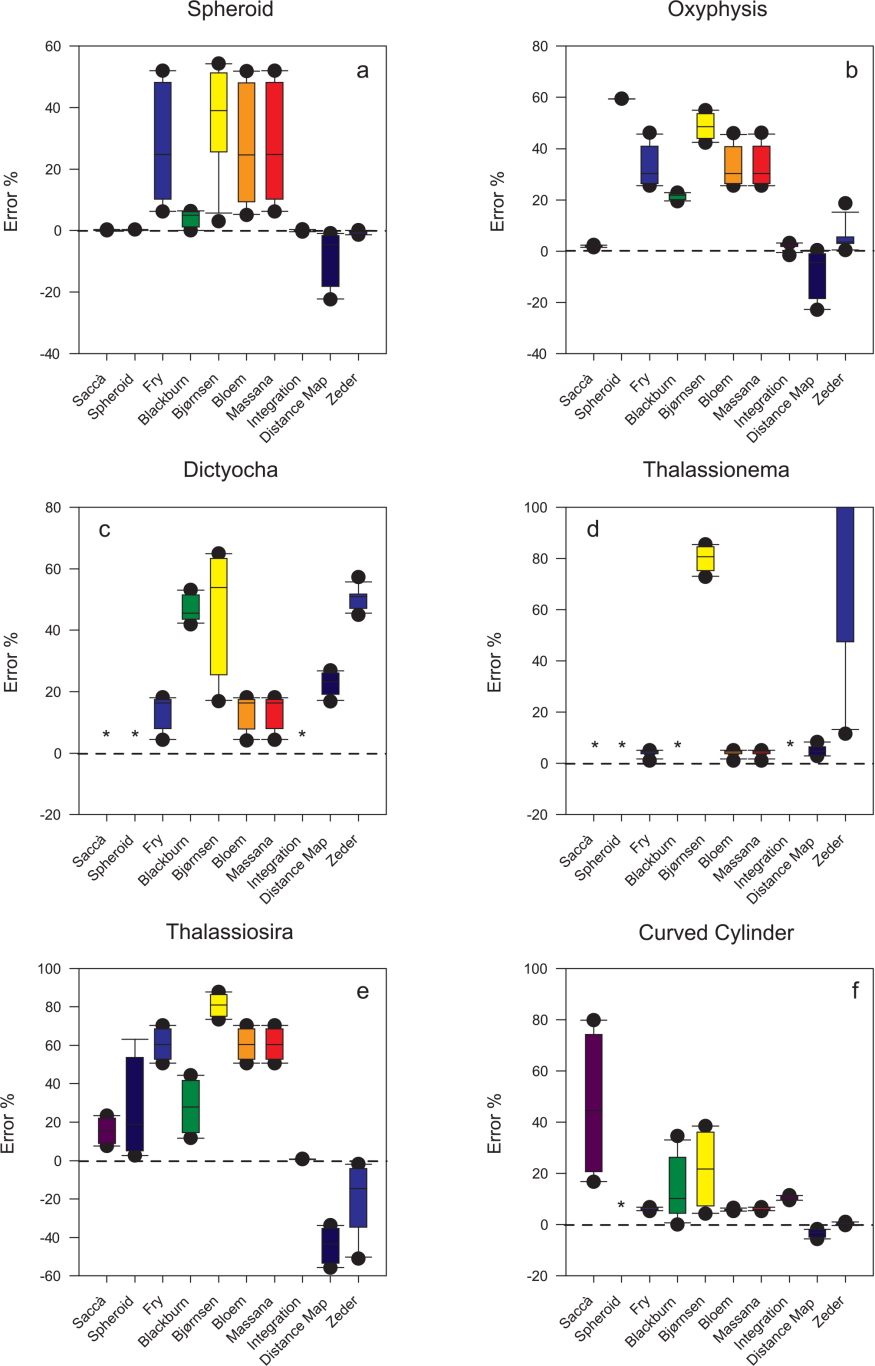
Box plots showing the errors caused by the new equation and the other tested methods in the estimation of the volume of the 3D shapes created with Cinema4D. The median, as well as the 10^th^, 25^th^, 75^th^, and 90^th^ percentiles are shown. Whiskers indicate the 10^th^ percentile and 90^th^ percentile, respectively, while the extent of outlying points (solid circles) identifies the data range (standard method is used to calculate percentile values). The * symbol denotes that values are off scale.

As for the *O*. *oxytoxoides* 3D reconstruction, independent of the aspect ratio, the prolate spheroid equation invariably yielded an overestimation of 59.44%, while the new method underestimated the actual volume by 1.46–2.35%, and the ‘integration’ method—and in particular, the implementation by Sosik—overestimated it by 1.63–2.49% (Fig 7B).

As expected, with the spheroidal shapes, the prolate spheroid equation was always exact, while the new method returned errors between -0.31 and +0.33%, and the ‘integration’ method between -0.37 and +0.37%. The algorithm proposed by Zeder was also remarkably precise: from -1.39 to +0.12% (Fig 7A). With the *O*. *oxytoxoides* shape, only Zeder and the ‘integration’ method yielded results comparable to those of the new method (Fig 7B), while the other tested methods returned different errors depending on the cell aspect ratio (Fig 7B). For the methods developed respectively by Fry, Blackburn and Bloem, only trivial differences were noticed between YABBA outcomes and the results of calculations made using FIJI measurements as input data for the respective equations. Hence, only these latter are reported here, for uniformity with the methods by Bjørnsen and Massana, which are not considered by YABBA. Indeed, the elaborated pre-processing performed by YABBA should have a minimal effect or no effect on clear-cut silhouettes such as those used here as input images. The ‘distance map’ algorithm systematically underestimated biovolume, with the magnitude of errors depending on the aspect ratio of the shape (Fig 7), as expected given the inherent assumptions. It should be noted that for these cases, the *convex area*/*area* proportions (1.16–1.18 for *O*. *oxytoxoides* and 1.00–1.01 for spheroids) were below the recommended limit (1.2) for the employment of the method [17]. With branched shapes having *convex area / area* proportions compatible with the ‘distance map’ algorithm, the latter was appreciably precise, returning errors between 2.83 and 8.39% for the *Thalassionema*-like shape, and between 16.79 and 26.87% for the *Dictyocha*-like shape; the new equation, the ‘integration’ method and, especially, the prolate spheroid equation, returned such exceedingly high errors as to be meaningless (they are out of scale in Fig 7C, 7D and 7F). Surprisingly, the methods proposed respectively by Fry, Bloem and by Massana returned considerably precise results with these branched shapes, even better than those obtained with the ‘distance map’ algorithm (Fig 7).

As shown in Fig 7F, the volume of curved cylinders with hemispherical ends was estimated quite reliably by Fry, Bloem and Massana (less than 7% underestimation), and also by Blackmore (below 11% overestimation). The ‘distance map’ algorithm underestimated the volumes by less than 6%, while the proposed equation yielded errors between +16% and +80% (Fig 7F).

A particular case was represented by the instance of *Thalassiosira*-like shapes, for which the ‘integration’ method gave the best results (errors: 0.64–0.87%), followed by the new method (7.56–23.38%). The prolate spheroid equation, Blackburn and Zeder returned, on average, moderate errors, but their performances were quite erratic, depending on the shapes’ proportions, while the ‘distance map’ algorithm was scarcely precise (errors between -33.65 and -55.75%), notwithstanding the relatively high *convex area / area* proportions (always above 1.2) (Fig 7E).

## Discussion

In plankton microbial ecology it is often necessary to estimate the biovolume of microorganisms from 2D micrographs. This is far from being straightforward, since the third dimension of a 3D object is typically obscured in 2D images. Most planktonic microorganisms, however, display an approximate radial symmetry, and the typical conditions under which images are acquired allow approximating these cells by solids of revolution. Most methods advised so far for the estimation of the biovolume of microorganisms rely on this basic assumption, and so does the equation proposed here. The latter proved to be remarkably reliable and stable with almost all shapes tested, except the branched ones (*Dictyocha*-like and *Thalassionema*-like).

The ‘distance map’ algorithm appears to be the best alternative for such kind of shapes, but the tests performed here confirm the conclusion [17] that it is not suitable for relatively ‘compact’ shapes (*area / convex area < 1*.*2*). The method recommended in this case [17] is the ‘integration’ algorithm [15], an improved implementation of which (Sosik, personal communication) is employed with the submersible imaging-in-flow instrument, Imaging FlowCytobot [23].

Notably, the proposed equation did better than the ‘integration’ method (including the improved version) with most tested shapes, except with the cylinders with hemispherical ends and the *Thalassiosira*-like shapes (which are largely composed of cylinders). Clearly, the ‘integration’ method gives better approximations of the cylindrical parts of these shapes but worse approximations of the spherical—or otherwise curved—ones, with respect to the new method. Additional tests on cylinders with hemispherical ends indicated that the error caused by the ‘integration’ algorithm decreased as the object became longer—that is, as the cylindrical part became more and more preponderant—while with the new method, the error increased, still remaining below 4% until an aspect ratio (*length / width*) of 6.36:1. Microorganisms hardly ever have precisely spherical caps, however, and further tests were designed to allow for different shape profiles. The volumes of the solids of revolution obtained from two analytic curves were estimated quite reliably by both the proposed method and the ‘integration’ algorithm, although the outcome of the latter was strictly dependent on the number and position of sample points.

The volume of a cylinder with hemispherical ends was estimated better with Fry, Blackburn and Massana than with the proposed equation. This is not surprising, however, as these methods assume a sphere or a cylinder with hemispherical ends as the cell shape, being them tailored on bacillus-like or coccus-like stereotypes of prokaryotes. On the other hand, the same methods gave worse volume estimations than did the new method with almost all other shapes; three notable exceptions are Fry, Bloem and Massana when they are applied to branched shapes such as the *Dictyocha*-like and *Thalassionema*-like ones. The reason for these outcomes is possibly that these methods are based on the cross-sectional surface area and perimeter, which are more stable and reliable measures than linear dimensions [3, 4, 24]. They are also clearly much more suitable for branched shapes, given the difficulty of extrapolating the length and width for these latter. Bjørnsen, on the other hand, yielded poorer and more erratic outcomes—although it is also based on area and perimeter—possibly because it employs an empirical equation that presumably relies on different assumptions [3]. Fry and Bloem returned quite reliable estimations, even of the volume of the curved cylinder, but they did not prove to be generally appropriate for shapes with high *convex area / area* proportions. The method by Blackburn, which employs the cross-sectional surface area and the longest cord measured, returned even worse estimations with the same shapes.

Although Zeder performed the best for the curved cylinder, it generally gave worse results than both the proposed equation and the ‘integration’ algorithm, especially for branched shapes. In fact, when the output visualisation option in YABBA was selected, inaccurate 3D reconstruction has often been observed. This depends on the fact that ‘the number of points reconstructed on the shape needs to be in an optimal range’ (Zeder, personal communication), and the present software version probably returned too ‘few points on the outline’. Hopefully, this will be fixed in future software versions, as this ingenious method is powerful and potentially suitable for a much wider variety of shapes than those for which it has been primarily designed.

The ‘distance map’ algorithm, set apart Fry and Bloem, was the most accurate method with branched shapes, and was also appreciably precise with the curved cylinder with hemispherical ends. Although errors were never excessively elevated, however, this algorithm was less reliable than expected. In fact, the full potential of the ‘distance map’ approach has not yet been exploited, as a representative width is currently used for the whole image instead of calculating each transect length individually. This would be demanding in terms of computational time and gets complicated near intersection regions [17], whereas the current solution, although not precise, is operationally adequate for second order shape correction (Sosik, personal communication).

While the proposed equation is not appropriate for branched or otherwise highly concave shapes—even those showing local rotational symmetry—it is easy to demonstrate that it is suitable for simple, curved shapes, provided that the length is measured along their curved rotational symmetry axis. Although good approximations may be obtained manually or semi-automatically, however, fully automatic methods for measuring curved lengths are susceptible to flaws. In fact, a process called ‘skeletonization’ [25, 26] is embedded in FIJI, and it has already been employed in the application WormSizer [27], designed for assessing size and shape of nematodes. Although it has worked well with fairly regular tube shapes like worms [27], and appears to be suitable for simple-shaped unicellular organisms (S5 Fig), problems arise with the wide range and differences in shapes found in plankton [17]. Moreover, this approach is too difficult and error prone to be implemented in a fully automated way (Sosik, personal communication).

In fact, it is probably impossible to estimate the volume of branched or highly concave shapes with a single equation, because their hidden dimensions cannot be inferred from a few basic parameters. For its stability with such shapes, the best alternative is, at present, the ‘distance map’ approach, particularly where local rotational symmetry can be assumed. A convenient solution for the estimation of the biovolume of a variety of plankton microorganisms is thus represented by a combination of the ‘distance map’ algorithm with the equation proposed here. A valid discrimination criterion could be the following:

$$1-\frac{Area}{Conv\;Area}>0.2$$ (11)

This is only slightly different from the coefficient already proposed for the ‘distance map’ and the ‘integration’ algorithms [17], but demonstrated more functional with *Oxyphysis* and *Thalassiosira*- like shapes. This criterion is not perfect, however, as *Thalassiosira*-like shapes, for example, were still often ascribed to the ‘distance map’ algorithm while, despite relatively high *convex area / area* proportions, they show strict rotational symmetry around a straight axis and, thus, would be best assessed either with the ‘integration’ method or the proposed equation. For the above reasons, no universal value could be found for this coefficient—or a possible alternative one—so a trade-off based on available data was chosen in order to minimise errors.

## Conclusion

The method described here is free from *a priori* geometrical assumptions—set aside global rotational symmetry—and is designed to take cell shape irregularities into account, resulting in remarkable precision and stability with most prokaryote and microbial eukaryote shapes. For its extreme simplicity, it requires neither elaborated pre-processing, such as contour tracing or shape recognition, nor redundant manual or semi-automatic measurements for each specimen. For these reasons, it is not computationally or labour intensive, and the entire procedure, from image acquisition to biovolume calculation, can easily be standardised and automated so as to be embedded within any image analysis software.

The proposed method is not suitable, however, for highly concave or branched shapes, such as those of many colony-forming species; also, its use with curved shapes is not straightforward. It should therefore be employed in combination with specific alternative solutions, if such morphologies are expected to be quantitatively important. At present, the best option to this purpose is the ‘distance map’ algorithm [17], whose only relevant assumption is local rotational symmetry. The results of this study show that the proposed equation is generally more reliable than the ‘integration’ approach, in addition to being less demanding in terms of computational resources. Thus, it may conveniently replace it in combination with the ‘distance map’ algorithm in automated cell imaging setups.

## Supporting Information

S1 Fig Representation of an ellipsoid inscribed in a cylinder, whose semi-axes are *a*, *b* and *c*. The base of the cylinder is an ellipse with semi-axes *a* and *b*, while the height of the cylinder is equal to *2c*.
(TIF)

S2 Fig Outlines of the medial cross-sections of the eight model shapes represented in Fig 1. The basic dimensions are indicated with relation to parameters *a* and *b*.
(TIF)

S3 Fig Cartesian representations of the 2D analytic functions whose rotation around the y-axis creates the two solids of revolution *a* and *b*. (EPS)

S4 Fig 3D model shapes created with the application Cinema4D and employed for the evaluation of the proposed equation in comparison with several other methods for biovolume estimation. (**a**) *Dictyocha*-like, (**b**) *Thalassiosira*-like, (**c**) prolate spheroid, (**d**) curved cylinder, (**e**) *Thalassionema*-like.
(TIF)

S5 Fig Basic steps in the measurement of the curved cell axis of a mixotrophic cryptophycean alga. (**a**) micrograph of the organism under phase contrast microscopy (total magnification 1000x) with ‘longest shortest path’ superimposed, (**b**) micrograph of the cryptophycean cell after image segmentation, (**c**) skeletonization of the segmented image, and (**d**) determination of the ‘longest shortest path’ of the skeleton.
(TIF)

S1 Table Synopsis of the methods/algorithms employed in this analysis. (DOCX)

## References

[1] Sieracki ME, Johnson PW, Sieburth JM. Detection, enumeration and sizing of planktonic bacteria by image analysed epifluorescence microscopy. Appl Environ Microbiol. 1985;49: 799–810. 2408564 10.1128/aem.49.4.799-810.1985 PMC238449

[2] Fry JC, Davies AR. An assessment of methods for measuring volumes of planktonic bacteria, with particular reference to television image-analysis. J Appl Bacteriol. 1985;58: 105–112.

[3] Bjørnsen PK. Automatic determination of bacterioplankton biomass by image analysis. Appl Environ Microbiol. 1986;51: 1199–1204. 16347077 10.1128/aem.51.6.1199-1204.1986 PMC239044

[4] Bloem J, Veninga M, Shepherd J. Fully automatic determination of soil bacterium numbers, cell volumes, and frequencies of dividing cells by confocal laser scanning microscopy and image analysis. Appl Environ Microbiol. 1995;61: 926–936. 16534976 10.1128/aem.61.3.926-936.1995 PMC1388375

[5] Massana R, Gasol JM, Bjørnsen PK, Blackburn N, Hagstrøm Å, Hietanen S, et al Measurement of bacterial size via image analysis of epifluorescence preparations: description of an inexpensive system and solutions to some of the most common problems. Scientia Marina. 1997;61: 397–407.

[6] Blackburn N, Hagström Å, Wikner J, Cuadros-Hansson R, Bjørnsen PK. Rapid determination of bacterial abundance, biovolume, morphology, and growth by neural network-based image analysis. Appl Environ Microbiol. 1998;64: 3246–3255. 9726867 10.1128/aem.64.9.3246-3255.1998 PMC106717

[7] Edler L. Phytoplankton and Chlorophyll: Recommendations on Methods for Marine Biological Studies in the Baltic Sea. 1979 Baltic Marine Biologists Publication No. 5.

[8] Rott E. Some results from phytoplankton counting intercalibrations. Schweiz Z Hydrol. 1981;43: 34–62.

[9] Carpentier CJ, Ketelaars HA, Wagenvoort AJ, Pikaar-Schoonen KCPR. Rapid and versatile routine measurements of plankton biovolumes with BACCHUS. J Plankton Res. 1999;21: 1877–1889.

[10] Hillebrand H, Dürselen CD, Kirschtel D, Pollingher U, Zohary T. Biovolume calculation for pelagic and benthic microalgae. Journal of Phycology. 1999;35: 403–424.

[11] Sun J, Liu D. Geometric models for calculating cell biovolume and surface area for phytoplankton. J Plankton Res. 2003;25: 1331–1346.

[12] Vadrucci MR, Cabrini M, Basset A. Biovolume determination of phytoplankton guilds in transitional water ecosystems of Mediterranean Ecoregion. Transit Water Bull. 2003;2: 83–102.

[13] Lyakh AM. A new method for accurate estimation of diatom biovolume and surface area. In: Kusber W-H, Jahn R, editors. Proceedings of the 1st Central European Diatom Meeting; 2007 March 23–25; Berlin-Dahlem, Germany. Berlin: Botanic Garden and Botanical Museum Berlin-Dahlem Freie Universität; 2007. p. 113–116.

[14] Lyakh AM. 3D-Microalgae software used for the estimation of microalgae biovolumes and surface area. Mod Phytomorphol. 2012;1: 89–91.

[15] Sieracki ME, Viles CL, Webb KL. Algorithm to estimate cell biovolume using image analyzed microscopy. Cytometry. 1989;10: 551–557. 2776571 10.1002/cyto.990100510

[16] Zeder M, Kohler E, Zeder L, Pernthaler J. A novel algorithm for the determination of bacterial cell volumes that is unbiased by cell morphology. Microsc Microanal. 2011;17: 799–809. doi: 10.1017/S1431927611012104 21910938 10.1017/S1431927611012104

[17] Moberg EA, Sosik HM. Distance maps to estimate cell volume from two-dimensional plankton images. Limnol Oceanogr Methods. 2012;10: 278–288.

[18] Smayda TJ. From phytoplankton to Biomass In: Sournia A, editor. Phytoplankton Manual. Monographs on Oceanographic Methodology 6 Paris: UNESCO; 1978 p. 273–279.

[19] Wetzel RG, Likens GE. Limnological Analyses. 2nd ed. New York: Springer-Verlag; 1991

[20] Benfield MC, Grosjean P, Culverhouse PF, Irigoien X, Sieracki ME, Lopez-Urrutia A, et al RAPID: Research on Automated Plankton Identification. Oceanography. 2007;20: 172–187.

[21] Verity PG, Robertson CY, Tronzo CR, Andrews MG, Nelson JR, Sieracki ME. Relationships between cell volume and the carbon and nitrogen content of marine photosynthetic nanoplankton. Limnol Oceanogr. 1992;37: 1434–1446.

[22] Sosik HM, Olson RJ. Automated taxonomic classification of phytoplankton sampled with imaging-inflow cytometry. Limnol Oceanogr Methods. 2007;5: 204–216

[23] Olson RJ, Sosik HM. A submersible imaging‐in‐flow instrument to analyze nano‐and microplankton: Imaging FlowCytobot. Limnol Oceanogr Methods. 2007;5: 195–203.

[24] Fry JC. Direct methods and biomass estimation. Method Microbiol. 1990;22: 41–85.

[25] Lee T, Kashyap RL, Chu C-N. Building skeleton models via 3-D medial surface/axis thinning algorithms. CVGIP: Graph Models Image Process. 1994;56: 462–478.

[26] Arganda-Carreras I. Skeletonize3D [Internet]. 2008. Available: http://imagejdocu.tudor.lu/doku.php?id=plugin:morphology:skeletonize3d:start.

[27] Moore BT, Jordan JM, Baugh LR. WormSizer: High-throughput analysis of nematode size and shape. PLoS One. 2013;8: e57142 doi: 10.1371/journal.pone.0057142 23451165 10.1371/journal.pone.0057142 PMC3579787

