# Supplementary figures and images for: A Simple yet Accurate Method for the Estimation of the Biovolume of Planktonic Microorganisms
Source: PLoS One. 2016 May 19;11(5):e0151955. doi: 10.1371/journal.pone.0151955 (PMC4873252; doi:10.1371/journal.pone.0151955)

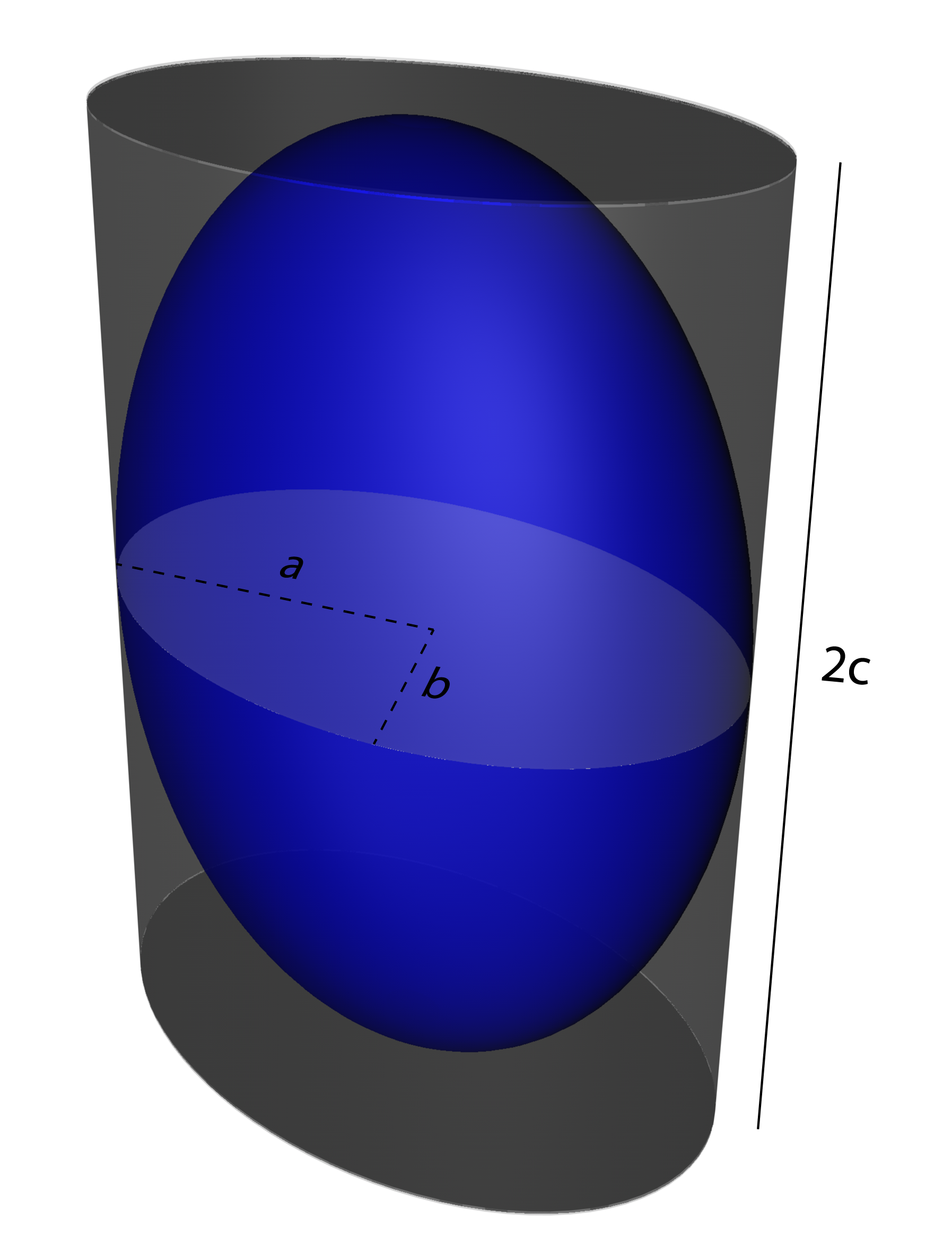

Supplement: S1 Fig — The base of the cylinder is an ellipse with semi-axes a and b, while the height of the cylinder is equal to 2c. (TIF) [file pone.0151955.s001.tif]

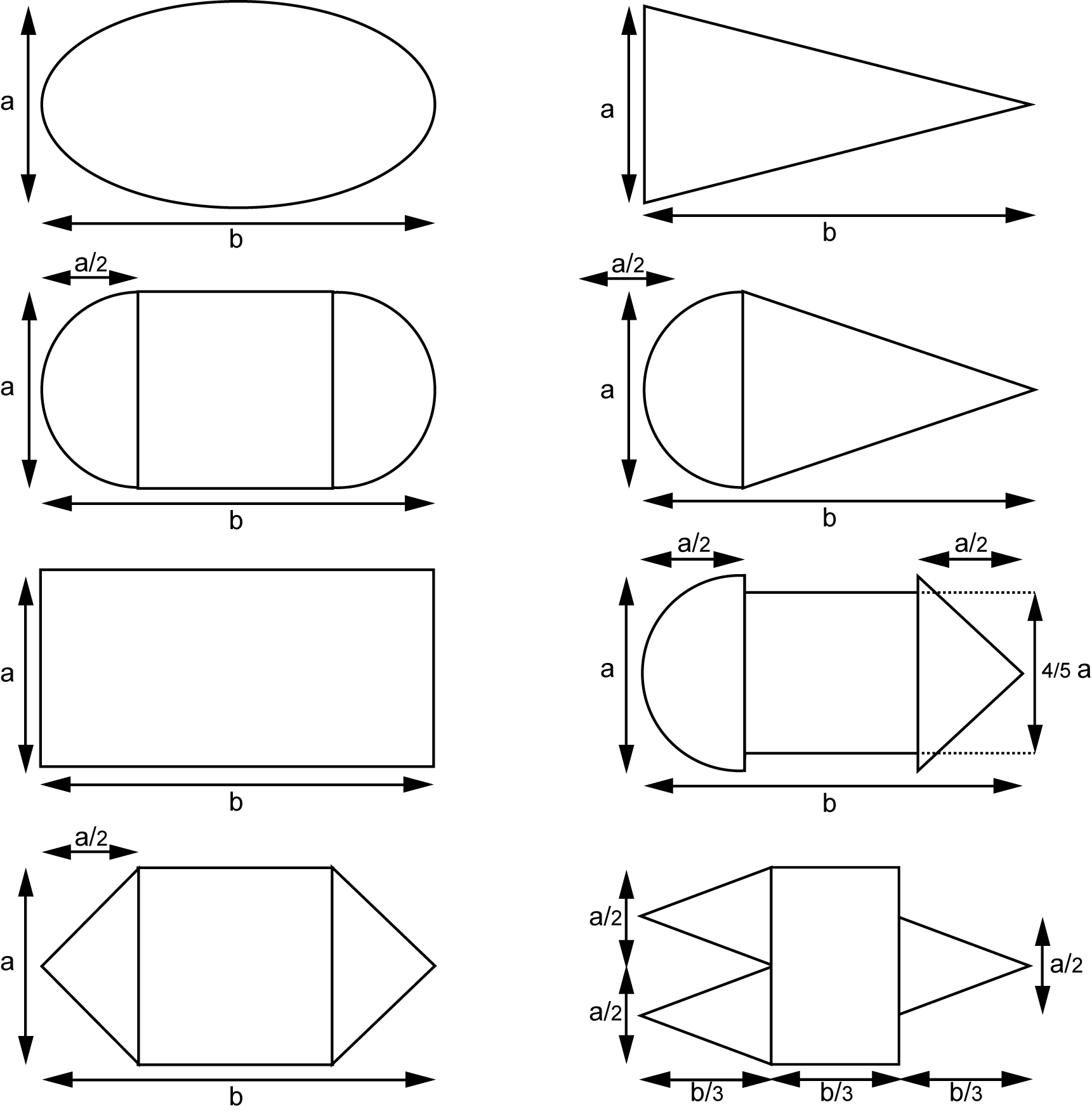

Supplement: S2 Fig — The basic dimensions are indicated with relation to parameters a and b. (TIF) [file pone.0151955.s002.tif]

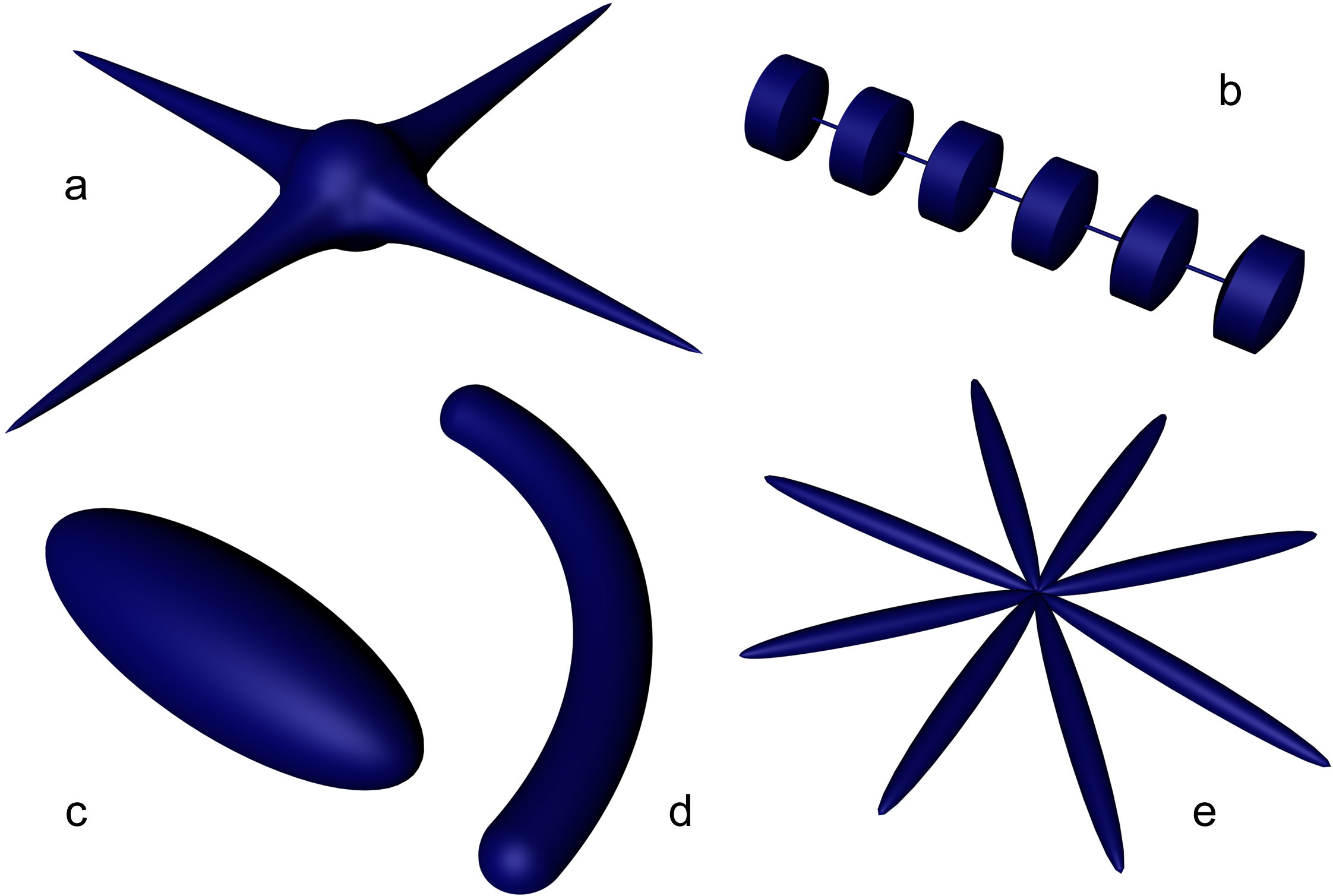

Supplement: S4 Fig — (a) Dictyocha-like, (b) Thalassiosira-like, (c) prolate spheroid, (d) curved cylinder, (e) Thalassionema-like. (TIF) [file pone.0151955.s004.tif]

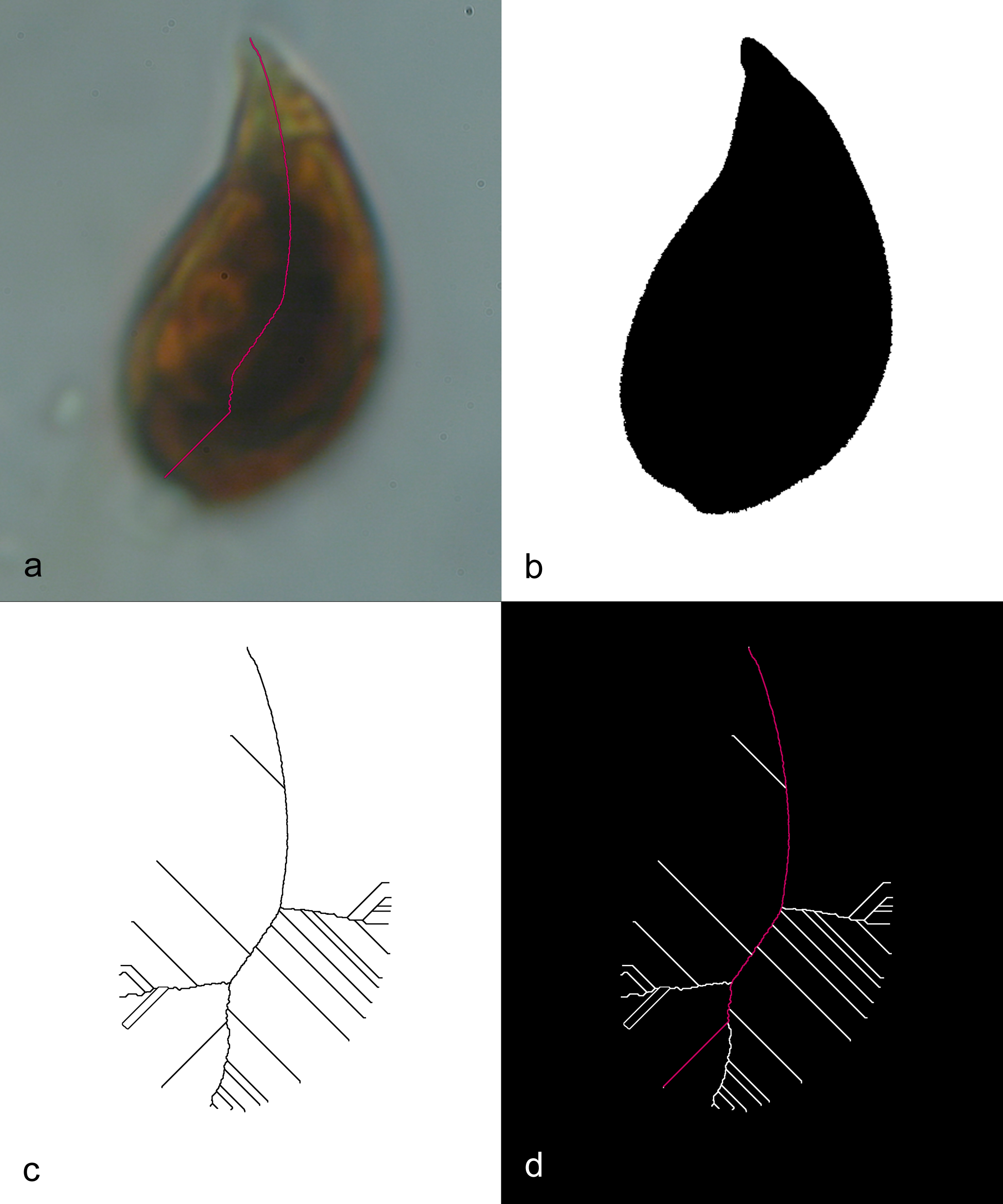

Supplement: S5 Fig — (a) micrograph of the organism under phase contrast microscopy (total magnification 1000x) with ‘longest shortest path’ superimposed, (b) micrograph of the cryptophycean cell after image segmentation, (c) skeletonization of the segmented image, and (d) determination of the ‘longest shortest path’ of the skeleton. (TIF) [file pone.0151955.s005.tif]
