# Supplementary material for: A Simple yet Accurate Method for the Estimation of the Biovolume of Planktonic Microorganisms
Source: PLoS One. 2016 May 19;11(5):e0151955. doi: 10.1371/journal.pone.0151955 (PMC4873252; doi:10.1371/journal.pone.0151955)
Supplement: S1 Table — (DOCX) [file pone.0151955.s006.docx]

**S1 Table. Synopsis of the methods/algorithms employed in this analysis.**

| METHODS | EQUATIONS/ALGORITHMS |
| --- | --- |
| Saccà (present paper) | $V=\frac{4}{3} A\sqrt{\frac{d A}{\pi l}}$ |
| Spheroid (e.g. Verity et al. 1992) | $V=\frac{4}{3}\pi\left( \frac{d}{2} \right)^{2}\frac{l}{2}$ |
| Fry (Fry and Davies 1985) | $d=\frac{\left( P^{2}-4\pi A \right)}{\pi}$  $l=\frac{P}{2}+\left( 1-\frac{\pi}{2} \right)d$  $V=\frac{\pi}{4}d^{2}\left( l-d \right)+\pi\frac{d^{3}}{6}$ |
| Blackburn (Blackburn et al. 1998) | $r=\frac{-l+\sqrt{l^{2}+A\left( \pi-4 \right)}}{\pi-4}$  $V=\frac{4}{3}\pi r^{3}+\pi r^{2}\left( l-2r \right)$ |
| Bjørnsen (Bjørnsen 1986) | $V=8.5 A^{2.5} {CP}^{-2}$ |
| Bloem (Bloem et al. 1995) | $F_{l}=\frac{P+\sqrt{\left( P^{2}-16A \right)}}{4}$  $F_{w}=\frac{P-\sqrt{\left( P^{2}-16A \right)}}{4}$  $V=\frac{\pi}{4}{F_{w}}^{2}\left( F_{l}-\frac{F_{w}}{3} \right)$  $V_{alt}= \frac{\pi}{6}{ECD}^{3}$  $ECD=2\sqrt{\frac{A}{\pi}}$ |
| Massana (Massana et al. 1997) | $w_{e}=\frac{P-\sqrt{P^{2}-4\pi A}}{\pi}$  $l_{e}=\frac{P}{2}+w_{e}\left( 1-\frac{\pi}{2} \right)$  $V=\frac{\pi}{4}{w_{e}}^{2}\left( l_{e}-\frac{w_{e}}{3} \right)$  $V_{alt}=\frac{4}{3}\sqrt{\frac{A^{3}}{\pi}}$ |
| Integration (Sieracki et al. 1998) | Algorithm implemented by Heidi Sosik (personal communication) |
| Distance Map (Moberg and Sosik 2012) | Algorithm described in Moberg and Sosik (2012) |
| Zeder (Zeder et al. 2011) | Algorithm described in Zeder et al. (2011) |

*l* = Cross-sectional length*

*d* = Cross-sectional width**

*A* = Cross-sectional surface area†

*P* = Cross-sectional perimeter†

*CP* = Cross-sectional convex perimeter‡

*F_l_* = Fiber length

*F_w_*= Fiber width

*w_e_* = Equivalent width

*l_e_* = Equivalent length

*r* = Equivalent radius

*ECD* = Equivalent circle diameter

*V* = Volume

*V_alt_* = Volume estimated through an alternative equation when primary equation is not workable

* Rotational symmetry axis has been employed for all geometric shapes, except *Ceratium*-like, which is not symmetric. Maximum Feret diameter (measured with the software FIJI-ImageJ) has been used for all model shapes reconstructed with Cinema4D. A geometrically determined maximum Feret diameter has been employed for the *Ceratium*-like shape.

** Maximum distance perpendicular to the rotational symmetry axis—or to the geometrically determined maximum Feret diameter—has been used for all geometric shapes. Minimum Feret diameter (measured with the software FIJI-ImageJ) has been employed for all model shapes reconstructed with Cinema4D.

† Cross-sectional surface area and perimeter have been calculated geometrically except for all model shapes reconstructed with Cinema4D, for which they have been measured with the software FIJI-ImageJ.

‡ Cross-sectional convex perimeter has been calculated geometrically for *Ceratium*-like and *Peridinium*-like shapes. For all model shapes reconstructed with Cinema4D, except the prolate spheroid model shape, it has been measured with the software FIJI-ImageJ.

Note: Cross-sectional convex perimeter, as well as cross-sectional convex area for the calculation of the discrimination coefficient

$$1-\frac{Area}{Conv. Area}>0.2$$

have been estimated with the software FIJI-ImageJ, subsequent to the application of the ‘convex hull’ macro plugin (<http://www.cse.unsw.edu.au/~lambert/java/3d/hull.html>). For all geometric shapes, except *Ceratium*-like and *Peridinium*-like, and for the prolate spheroid model shape reconstructed with Cinema4D, cross-sectional convex perimeter and area were equal respectively to conventional perimeter and area.
